# Can Monetary Reward Modulate Social Attention?

**DOI:** 10.3389/fpsyg.2018.02213

**Published:** 2018-11-14

**Authors:** Emanuele Lo Gerfo, Jacopo De Angelis, Alessandra Vergallito, Francesco Bossi, Leonor Josefina Romero Lauro, Paola Ricciardelli

**Affiliations:** ^1^Department of Economics, Management and Statistics, University of Milano-Bicocca, Milan, Italy; ^2^CISEPS, University of Milano-Bicocca, Milan, Italy; ^3^NeuroMI – Milan Center for Neuroscience, Milan, Italy; ^4^Department of Psychology, University of Milano-Bicocca, Milan, Italy; ^5^Social Cognition in Human-Robot Interaction, Istituto Italiano di Tecnologia, Genoa, Italy

**Keywords:** social attention, orienting of attention, gaze cueing effect, monetary reward, social cognition

## Abstract

Selective visual attention is a primary cognitive function, which allows the selection of the most relevant stimuli in the environment by prioritizing their processing. Several studies showed that this process can be influenced by both social signals, such as gaze direction (i.e., the Gaze Cueing Effect, GCE) and by the motivational valence of gratifying stimuli, such as monetary rewards. The aim of this study was to explore whether GCE could be modulated by a monetary reward. To this end, we created an experiment in which participants performed a gaze cuing task before and after an implicit learning task aiming to induce an association between gaze direction and monetary reward (experimental condition), or after a perceptual task (control condition). Statistical analyses were conducted following both a frequentist and a Bayesian approach. Results supported previous findings showing the presence of the GCE, i.e., faster responses in congruent trials when the target appeared in the gazed-at location. Interestingly, our results did not reveal significant differences among the conditions. Therefore, contrary to what was reported by previous attentional orienting studies with non-social stimuli, monetary reward does not seem to be able to modulate (or interfere with) the orienting of attention mediated by gaze direction as measured by the GCE. Taken together our results suggest that social signals such as gaze direction have a greater impact than monetary reward in orienting selective attention.

## Introduction

Selective attention is a primary cognitive process, which allows the selection of the most relevant stimuli in the environment by prioritizing their processing (Desimone and Duncan, 1995; Pashler, 1998; Reynolds and Chelazzi, 2004). This selection has been demonstrated to rely both on bottom-up factors, such as perceptual features of the stimuli (Yantis and Jonides, 1984; Theeuwes, 1992; Ando, 2002), and on top-down factors, such as individual goals (Kristjánsson 2006; Kristjánsson and Campana, 2010), the context in which stimuli are embedded (Wolfe, 1994; Egeth and Yantis, 1997; Chelazzi, 1999) and previous experience (Kristjánsson and Campana, 2010; Chun, 2011; Awh et al., 2012).

Several studies focused on the role played by social signals such as eye gaze direction in orienting selective attention (Ricciardelli et al., 2000; Frishen et al., 2007). Social attention, namely the tendency to attend the same object that another person is looking at (Frishen et al., 2007), has received much consideration by scholars. This attentional orientation, specific to humans and other primates, has been also defined “*joint attention*” (Bruner, 1983) and emerges in children starting from 2 months (Maurer, 1985; Baron-Cohen, 1994). Following another person’s gaze toward specific regions of the environment provides the observer with considerable information, both in terms of the saliency of the co-attended stimuli and providing cues on the looker’s mental states. Indeed, this ability has a strong adaptive valence since it can communicate the presence of possible danger or threat (Menzel and Halperin, 1975; Byrne, and Whiten, 1991).

### Eye Gaze as a Special Cue to Allocate Visual Attention

In experimental settings, modifications of Posner’s cueing paradigm (Posner, 1980) have usually been employed to study the attentional shifts triggered by the observation of eye-gaze direction (Frishen et al., 2007), namely the so-called gaze cueing paradigm (Friesen and Kingstone, 1998; Driver et al., 1999). In this paradigm, participants are required to identify target letters appearing to the left or to the right of a face placed at the center of the screen. Unlike Posner’s paradigm, the gaze direction of a face looking either to the left or right direction substitutes the central arrow cue typically used in traditional attentional paradigms. Previous research has demonstrated that reaction times (RTs) are significantly faster in congruent-cue trials (i.e., with the target appearing in the gazed-at location) than in incongruent-cue ones (Friesen and Kingstone, 1998; Frishen et al., 2007) even when gaze direction is task-irrelevant. This attentional facilitation emerges with relative early stimulus onset asynchrony (SOA) (105–300 ms, Perez-Osorio et al., 2017) and disappears with longer SOA (1005 ms) (Friesen and Kingstone, 1998). This effect is known as the Gaze Cueing Effect (GCE) and has been replicated by many other studies (Hamilton, 2016; Ulloa et al., 2018; for a review see Frishen et al., 2007).

However, a recent study by Takao et al. (2018) showed that GCE might be influenced by cultural differences. In particular, the authors reported that in Western people the GCE emerges both at short and long SOAs (117 ms vs. 700 ms), whereas Japanese participants only show GCE at shorter SOAs. Traditionally in Posner’s paradigm, two types of cues can be used to induce the orientation of visual spatial attention, namely *endogenous* and *exogenous cues*. The endogenous cue usually consists of an arrow appearing at the center of the screen above or below a central fixation point. It provides participants with explicit information about the target possible location (i.e., participants expect that the target is more likely to appear at the cued location) and requires a voluntary orientation of visual spatial attention.

By contrast, the exogenous cue consists of a salient event (e.g., a flashing light) appearing in the periphery of the screen. Such a cue, by virtue of its visual saliency, automatically orients attention to the cued location, even if it does not inform about where the target will appear and, therefore, is task-irrelevant (Posner, 1980; Posner and Cohen, 1984).

Concerning GCE, it is still a matter of debate whether it can be considered the result of an endogenous (voluntary) or exogenous (automatic) orientation. Indeed, at least two key criteria need to be present in order to consider a certain process as “automatic” (for a review see Santangelo and Spence, 2008). First, automatic processes are immune to the interference of a concurrent task and of its cognitive load (*load-insensitivity criterion*). Second, automatic processes are not influenced by the individual’s intentional control (*intentionality criterion*).

Early studies suggested substantial similarities between spatial cueing induced by gaze direction and the one triggered by exogenous cues, suggesting that both produced significant orienting even when spatially uninformative or counter-informative. Initially, such evidence has been taken to support the automatic nature of GCE (Friesen and Kingstone, 1998; Driver et al., 1999).

Recently, however, scholars suggest that GCE can be influenced by top-down processes such as contextual cues, knowledge and expectation, and reading of other’s mind (Bayliss et al., 2010; Teufel et al., 2010; Wiese et al., 2012, 2013, 2014; Ehrlich et al., 2014; Wykowska et al., 2014; Cole et al., 2015; Perez-Osorio et al., 2017; Hayward and Ristic, 2018).

Regarding the load-insensitivity criterion, it is still debated whether GCE can be modulated by increasing concurrent tasks cognitive loads: some authors posited that increasing working memory demands do not interfere with GCE effects (e.g., Xu et al., 2011; Hayward and Ristic, 2013), whereas others found some interference (Bobak and Langton, 2015).

Authors also highlighted differences between using arrows or gaze as triggers to drive attention (Driver et al., 1999; Friesen and Kingstone, 2003; Friesen et al., 2004), suggesting that orienting attention toward somebody else’s gaze direction could be considered a special type of attentional orienting, due to the strong biological and social significance of gaze.

On the other hand, some evidence questioned these conclusions by showing that gaze and arrows would instead induce similar behavioral effects (Tipples, 2008; Guzzon et al., 2010).

Far from a conclusive definition of the nature of GCE, it seems possible to conclude that it is not a strictly automatic process, since it is not immune to top-down modulation and is somehow similar to the effect elicited by social over-learned cues such as arrows (Brignani et al., 2009; Guzzon et al., 2010).

### Reward and Selective Attention

Previous evidence suggests that reward is capable of modulating selective attention (Chelazzi et al., 2013). Similar to the *law of effect*, which suggests that an action followed by satisfying effects become more likely to reoccur than an action that produces discomfort (Thorndike, 1911), attentional processes are subject to mechanisms that re-modulate attentional focus toward specific items or spatial location based on previous outcomes. The memory system underlying this re-modulation of attentional processes strengthens the trace of items with high reward outcomes more than items with poor consequences (Della Libera and Chelazzi, 2009).

Specifically, behavioral findings revealed that monetary reward significantly improves detection performance in spatial attentional tasks by acting as an incentive to the participants’ performance (Engelmann and Pessoa, 2007; Engelmann et al., 2009; Pessoa and Engelmann, 2010). Interestingly, although this effect has been found to be stronger for congruent trials, RTs significantly decrease also in incongruent conditions with the increase of monetary reward (Engelmann et al., 2009). According to the authors, this evidence suggests that the motivational cue induced by the monetary reward impairs cue processing, or as an alternative explanation, it could facilitate the disengagement from an incongruent cue location (Engelmann et al., 2009). A similar effect was recently found by Bourgeois et al. (2016, 2017), who suggested that high rewarding stimuli may mitigate the typical facilitation induced by both exogenous and endogenous cues in visual search tasks, also when these stimuli are task-irrelevant. Moreover, these findings are consistent with the hypothesis that the motivational valence of visual stimuli may act as a strong exogenous signal, solving competition among different stimuli and guiding visual search behavior (Anderson et al., 2013; Chelazzi et al., 2014).

Chelazzi et al. (2014) also examined the alteration of high and low rewarded spatial priority maps, which authors defined as “real-time representations of the behavioral salience of locations in the visual field,” in a cross stimulus-competition paradigm (for an exhaustive description of the task see Chelazzi et al., 2014). Their behavioral paradigm comprised a baseline session, followed by a learning phase and a final test session. During the learning session, participants performed a visual search task on a target among distractors, in which correct responses in certain spatial locations were associated with high reward and others with low reward.

The results showed that in the test session, compared to the initial baseline, a target presented at a high-reward location increased its priority when paired with a target at a low reward location, and vice-versa. The authors concluded that reward was able to modulate spatial attention inducing plastic changes in the priority maps of space.

Recent evidence reported how reward-based implicit learning processes may affect the motivational valence of a stimulus, by influencing the deployment of attentional resources on features or locations able to optimize the organism adaptation to the environment and to favor positive behavioral outcomes (Chelazzi et al., 2013). For instance, Anderson et al. (2014) found that only target features (i.e., color) carrying information about subsequent reward magnitude, rather than the mere stimulus features *per se*, predicted attentional capture by those features (Anderson et al., 2014).

Specifically, two separate reward-related learning mechanisms have been identified. The first mechanism concerns conditions in which reward is perceived as a feedback on performance and consequently involves the cognitive monitoring of the individual on his/her performance (O’Doherty, 2004; Schultz, 2006). The second one, instead, takes into account conditions in which reward relates to random events characterizing task performance, where the individual produces associations between objects in the environment and the reward accompanying them (for a review see Chelazzi et al., 2013).

Other studies, based on the classical conditioning paradigm (Pavlov, 1927; Dayan et al., 2000), suggest a third learning mechanism, allowing the passive presentation of the pairing between a certain stimulus and a reward to be learnt. This happens even without awareness of the stimuli or of the contingence between stimulus and reinforcement (Büchel et al., 1998; Pessiglione et al., 2008; Seitz et al., 2009).

It is worth noting that reward influence on attentional orienting and implicit learning seems to last for a long time, by leaving long-term memory traces of the rewarding stimulus (Della Libera and Chelazzi, 2009).

Concerning the neural correlates involved in reward effects on visual attention, neuroimaging studies have highlighted a complex neural network. This includes regions which have been found to be activated during attentional tasks (i.e., frontal eye field, anterior cingulate cortex, intraparietal sulcus and temporo-parietal junction), visual stimuli processing (i.e., occipito-temporal visual cortex sites) and reward (i.e., caudate; orbitofrontal cortex, nucleus accumbens) (Engelmann et al., 2009; Pessoa and Engelmann, 2010). Accordingly, Pessoa and Engelmann (2010) suggested a model in which motivation and attention are “integrated” in affecting attentional behavior and modulated by the activation of a common core of regions involved both in visual attention orienting and in determining the rewarding valence of stimuli.

### Aims of the Present Study

As previously mentioned, studies that investigated the motivational role of reward presentation in guiding visual attention orientation for non-social stimuli did not explore a possible interaction between GCE and reward processing. Specifically, no studies tested whether and how monetary reward can modulate the effect of social cues on attention orienting, given that both types of cues (gaze direction and rewarded spatial location) could automatically orient visual attention. To this end, the current study aimed to investigate the role played by monetary reward in modulating social attention.

As in Chelazzi et al. (2014) we created an experimental procedure that was divided in three phases: a *baseline phase*, consisting of a gaze cueing task; a *learning phase*, in which we created an implicit association between gaze direction and monetary reward; a *test phase*, which evaluated the effect of reward on GCE, in which the gaze cueing task was presented again. Crucially, in the baseline and test phases, reward was not used.

Our prediction was that the association between gaze direction and monetary reward, learnt during the implicit learning task, would strengthen the allocation of attention only toward the location rewarded by the direction of gaze. According to this hypothesis, we expected to find a larger GCE for the rewarding than non-rewarding gaze direction.

## Materials and Methods

### Participants

60 Italian students (female 37, mean age = 23.61) of the University of Milano - Bicocca were randomly assigned to two experimental conditions. Another 30 students (19 female; mean age = 23.12) took part in a control condition. All participants were right-handed with no history of neurological or psychiatric diseases, had normal or corrected-to-normal vision and were naïve as to the experimental purpose. They received 5 Euros for their participation.

The participants gave their written informed consent before starting the experiment. The study was conducted in accordance with the ethical standards laid down in the 2013 Declaration of Helsinki and fulfilled the ethical standard procedure recommended by the American Psychological Association (APA).

The experiment was approved by the Ethics Committee of the University of Milano-Bicocca.

### Procedure

The experiment was set up and carried out in the Experimental Psychology Lab of the Department of Psychology at the University of Milano-Bicocca. Stimuli presentation and responses registration were controlled through the software E-Prime 2.0 Professional (Psychology Software Tools^1^). The participants sat approximately 57 cm away from a 15-inch LCD monitor. A standard Italian keyboard was used to register participants’ responses. The experiment was conducted in a dark room without windows.

The experiment included three sessions: an initial baseline session, with a gaze cuing task; an implicit learning session, in which a certain gaze direction was always associated with a reward; a test session, which was identical to the baseline.

In the control condition, the implicit learning session was replaced by a simple perceptual task, and no reward was delivered.

#### Gaze Cueing Task

This task was performed by the participants both during the baseline and test sessions. A gray scale photograph (6.2° × 1.7°) of the eye region of a female face was used as the gaze cue. In detail, three photographs versions were used: one with straight gaze, one with the gaze averted leftwards and one with the gaze averted rightwards.

The targets consisted of two capital letters, L or T (0.97°), which could appear on the right or on left sides (6.84°) of the screen. The participants were instructed to press on the keyboard with the right middle finger and the right annular, respectively, the V-key, whenever the letter L appeared on the left side of the screen, and the B-key when the letter L appeared on the right side of the screen (keys was covered by brown and pink paper squares, respectively). No response was required when the letter T appeared. We used a go/no-go task in order to increase task difficulty and investigate whether reward reduced response inhibition toward the reinforcing gaze directions or rewarded space locations.

**FIGURE 1:**
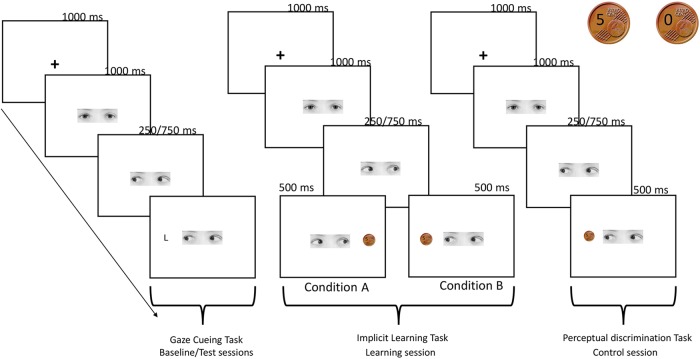
Schematic procedure of the experiment. *Gaze cueing task:* a fixation cross appeared for 1000 ms at the center of the screen then a face-cue looking straight was presented and 1000 ms after the onset, randomly gazed toward left or right. An upper-case letter (L or T) appeared after 250 or 750 ms (SOA) with the same probability at the right or at the left side of the screen. Participants pressed two different keys on the keyboard, depending on whether the target L appeared on the left or right side of the screen. No response was required when the letter T appeared. *Implicit learning* *task:* trial sequence of events was the same of the gaze cueing task, except for the target presentation. In this case, 1 s after the gaze shift, a coin indicating the monetary reward (5 cents) or the no-reward (0 cents) appeared in one of the two sides of the screen. In condition A participants were aware that they were receiving a reward of 5 euro cent in the 100% of trials in which gaze direction turned on the right, while when the gaze was directed to the left they were not receiving a reward in the 100% of trials (the two coins used in the reward and control tasks are showed in the right top of the figure). The reverse pattern was true for condition B. Participants were asked to predict the reward presentation. *Perceptual discrimination task:* trial sequence of events was the same of the implicit learning task, but in this control condition coin’s presentation was not associated to a reward. Participants were asked to predict if the picture of the coin contained either the number 5 or the number 0.

The experimental session comprised 256 trials that were split into two blocks of 128 trials each, with a break in between. In each trial, the sequence of events was as follows: (1) a fixation cross appeared for 1000 ms at the center of the screen; (2) a gaze-cue looking straight was presented and 1000 ms after its onset, randomly gazed toward the left or the right; (3) an upper-case letter (L or T) then appeared at the right or at the left side of the screen either after 250 or 750 ms (SOAs) with the same probability (6.84°, see Figure 1 for a schematic representation of the procedure). The target letter could appear in the location cued by the gaze (congruent trials) or on the opposite side (incongruent trials). RTs and accuracy were recorded.

We chose to implement a short (250 ms) and a long (750 ms) SOA because we wanted to be sure of finding a robust GCE effect, considering that there is no clear consensus on the optimal presentation timing (Friesen and Kingstone, 1998; Perez-Osorio et al., 2017; Takao et al., 2018).

#### Implicit Learning Task

This task comprised 200 trials, i.e., 100 trials of gain and 100 trials of non-gain. At the beginning of the session, participants were informed that they were starting a monetary exchange with another participant, called Participant B, who could decide to split 10 cents between himself and the participant, or to keep for her/himself the entire amount of money. In reality, the procedure was entirely controlled by a pc custom-program. The participants were asked to guess Participant B’s choices so as to keep their attention focused on the entire procedure. The participants were told that Participant B’s choice was independent from their guess. In fact, Participant B’s decision of delivering or not some money (gain or no-gain) was communicated by the direction of the gaze cue, which could shift toward the right or the left side of the screen.

We chose this procedure to create an association between gaze direction and the delivery of monetary reward, thus making it a conditioned stimulus. The key point of our procedure, in fact, was to give to the averted gaze a monetary value through an implicit learning procedure along with (on top of) its intrinsic (or well-established) social meaning and value.

The participants were randomly assigned to two conditions: (condition A – *N* = 30, female = 19; condition B – *N* = 30, female = 18) in which the earning was associated with a rightward or leftward gaze shift, respectively. More specifically in condition A, participants were told that they would receive a reward in all the trials in which the gaze was shifted to the right, and no reward in all the trials in which the gaze was shifted to the left. The reverse pattern was true for condition B.

The trial sequence of event was the same to the one described for the gaze cueing task, except for the target presentation. In this case, 1 s after the gaze shift, a coin indicating the monetary reward (5 cents) or the no-reward (0 cents) appeared in one of the two sides of the screen. In order to create two coins which look the same, a real photo of a 5-euro-cents coin was modified with an imaging manipulation program (GIMP 2.8), writing on it the number 5 and number 0 (Figure 1). At the end of each trial, a sentence on the screen summarized the total amount of money that the participants had won up until that trial. Concerning the prediction task, in group A the participants were required to press with the right middle finger the H-key on the keyboard when they expected to win, and with the right annular the N-key when they expected not to win, whereas in group B the participants were instructed to do the opposite.

It is to be noted that in our experiment, the reward was not associated with the participant’s choice or performance. By contrast, the explicit association between gaze direction and the delivery of reward was supposed to be implicitly reinforced by the participants based upon the stimulus contingencies, as in the classical conditioning procedure (Pavlov, 1927; Büchel et al., 1998; Pessiglione et al., 2008; Seitz et al., 2009). At the end of the experiment, the participants received the same amount of money (5 Euros).

#### Perceptual Discrimination Task

This task was performed only by the participants who took part in the control condition. It comprised 200 trials. The structure of the task was the same of the one used in the implicit learning task (see Figure 1). At the beginning of the task, the participants were informed that they were starting a game, in which they had to predict the moves of a second virtual player, called Participant B. Specifically, Participant B could decide to show a picture of a coin containing either the number 5 or the number 0. The side of the coin presentation was balanced between participants, with half of participants seeing the 5 coin when the gaze turned left and the other half seeing it when the gaze turned right. This procedure was chosen to exactly match the stimuli and the structure of the implicit learning task administered to the participants assigned to conditions A and B. Crucially, in this case, the coin’s presentation was not associated with a reward.

During the task, the participants were required to predict Participant B’s choices (i.e., number 5 vs. number 0 coins), which were indicated by the gaze shifting toward the right or the left side of the screen. The participants made their prediction by pressing the H or N-keys (with the right middle finger and the right annular, respectively), which were covered with blue and yellow paper squares.

### Data Analysis

The data of four participants were lost due to an overwrite error. One participant had a high rate of incorrect response ( >35%) and was excluded from the analysis. We report here the number of participants analyzed for each condition: condition A, 28 participants (18 female); condition B, 25 participants (15 female); control condition 30 participants (19 female).

For each participant and each condition, the mean and standard deviation (SD) of correct trials were computed. RTs slower and faster than 2 SD from individual mean were removed and excluded from subsequent analyses. With this procedure the 6,51% of trials was removed. Then for each participant and each condition [congruency (2), SOA (2), condition (2) and side (2)] a mean value of RTs was calculated.

An analysis of errors, i.e., when the participants responded to the no-go trials (with the target “T”), was not carried out because the percentage of these responses was very low (4.83%).

The RTs data for the correct responses were analyzed using JASP software (JASP Team, 2018), an open-source, simple and user-friendly R-based software aimed to run both frequentist (classical) and Bayesian analyses. As explained below, Bayesian analyses bring some important benefits when combined with the results of frequentist analyses.

Unlike frequentist statistical analyses, the output of a Bayesian analysis is typically the Bayes Factor (BF). The BF shows how likely data are to arise from one model, compared to another one (Wagenmakers et al., 2017). Typically, the two models are: a null model, predicting the null hypothesis (H_0_, i.e., the absence of an effect of the parameter); a second model predicting the alternative hypothesis (H_1_, i.e., an effect of the parameter). Therefore, the BF reflects the ratio between the likelihood of the data given H_0_ and the likelihood of the data given H_1_. In other words, the higher the BF, the more likely are the data given one of the two hypotheses. An important difference between Bayesian and frequentist statistics is the fact that a *p*-value reflects the likelihood of the data given H_0_. The likelihood of the data given H_1_ is not factored into the *p*-value, whereas it is factored into the BF. Frequentist statistics allows us to reject (or not) the null hypothesis, while Bayesian statistics allows us to evaluate and quantify the evidence in favor of H_0_ or H_1_.

The prior distribution of the data was set as a non-informative prior (*r* scale fixed effect = 0.5, *r* scale random effects = 1, *r* scale covariates = 0.354), since we had no specific *a priori* information. This setting corresponds to JASP default settings for repeated measure ANOVA, as recommended by the software programmers (Wagenmakers et al., 2017).

The BFs related to the effects were computed using a method suggested by Sebastiaan Mathôt (Wagenmakers et al., 2017; JASP Team, 2018), which takes into account the probability of different models given the data [i.e., P(model| data)]: the probability of models containing the effect of interest is compared to the probability of equivalent models stripped of the effect. Higher order interactions are excluded. This method is referred to as “Effects across matched models” output in JASP. Several ANOVAs, reported below, were then performed using this method.

The first two ANOVAs aimed to investigate the presence of GCE in the baseline session (before any manipulations), using both classical (Analysis 1) and Bayesian hypothesis testing (Analysis 2). Both analyses took into account two within-subject independent factors: congruency (2 levels: congruent vs. incongruent) and SOA (2 levels: 250 vs. 750 ms); and one between-subject factor: conditions (3 levels: condition A, condition B, and control condition), in a full factorial model. The dependent variable was RTs averaged across all correct trials in each condition.

The third and fourth ANOVAs aimed to investigate the effect of monetary reward on the GCE, using both classical (Analysis 3) and Bayesian hypothesis testing (Analysis 4). We created a factor named *reward* with three levels: (1) *rewarding gaze direction*, namely right (condition A) or left (condition B); (2) *no rewarding gaze direction*, namely left (condition A) or right (condition B); (3) *control condition*, in which no reward was delivered.

Both analyses took into account three within-subject independent factors: time (2 levels: baseline vs. test session), SOA (2 levels: 250 vs. 750 ms), congruency (congruent vs. incongruent trials) and one between-subject factor: reward (3 levels: rewarding gaze direction vs. no rewarding gaze direction vs. control condition), in a full factorial model. The dependent variable was the RTs averaged across all correct trials in each condition.

#### Results

##### ANOVA 1: with frequentist approach

Results from the first analysis showed a main significant effect of congruency, SOA and conditions: [congruency: *F*(1,82) = 32.535, *p* < 0.001, $\eta_{p}^{2}$ = 0.284; SOA: *F*(1,82) = 82.491, *p* < 0.001, $\eta_{p}^{2}$ = 0.501; conditions: *F*(2,82) = 5.893, *p* = 0.004, $\eta_{p}^{2}$ = 0.126]. All two- and three-way interactions were not statistically significant (all *F*s < 2.1, all *p*s > 0.14).

The main effect of congruency was due to faster RTs for congruent trials (mean = 421.28 ms, std dev = 45.16) than for incongruent trials (mean = 433.49 ms, std dev = 48.79). The main effect of SOA showed faster RTs for the 750 ms SOA (mean = 418.26 ms, std dev = 48.74) than for the 250 ms SOA (mean = 436.50 ms, std dev = 45.22). *Post hoc* comparisons (Bonferroni-corrected) performed on the main effect of condition highlighted that the participants assigned to the control condition (mean = 406.31 ms, std dev: 35.03) had shorter RTs than the participants assigned to conditions A and B (condition A: mean = 436.26 ms, std dev: 54.54, *t* = 2.725, *p* = 0.024; condition B: mean = 441.79, std dev = 45.45, *t* = 3.137, *p* = 0.007), while condition A and B did not differ from each other (*t* = -0.485, *p* > 0.999).

##### ANOVA 2: with Bayesian approach

Results of this analysis showed the following effects: congruency: BF_10_ = 2.051e + 7; SOA: BF_10_ = 1.304e + 16; conditions: BF_10_ = 9.029. All the interaction effects showed BF_10_ < 0.4. Therefore, we can conclude that, with regards to the effects of congruency, SOA and conditions, data were much more likely to be due to the alternative than the null hypothesis (i.e., data were explained in an extremely complete way by these effects). On the other hand, data were more than twice as likely to occur given the null than the alternative hypothesis on all the interaction effects. These results showed anecdotal to moderate evidence for H_0_ (Lee and Wagenmakers, 2013), indicating that the effect of congruency (i.e., GCE) was most probably not influenced by the effects of SOA, conditions, or the interaction between them.

##### ANOVA 3: with frequentist approach

**FIGURE 2:**
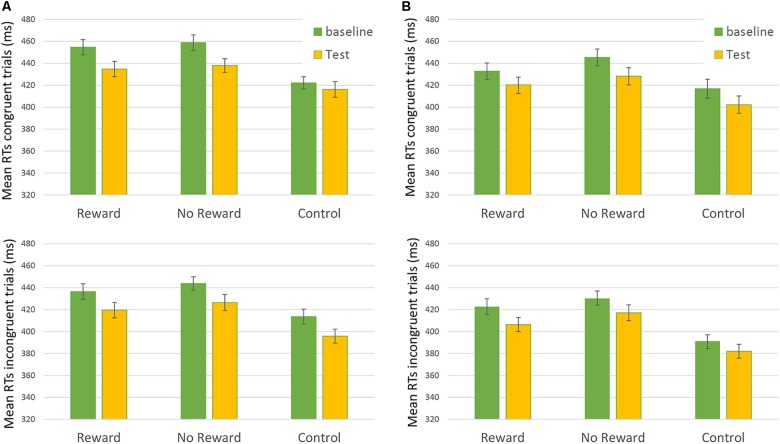
Mean RTs of Congruent (upper) and Incongruent (lower) trial in the “Rewarded,” “No-rewarded,” and “Control” conditions in baseline and Test session. On the left of the figure **(A)** 250-ms SOA are represented, on the Right **(B)** 750-ms SOA are shown. Bars represent the standard error.

Results from the third analysis demonstrated that the time ^∗^ reward ^∗^ congruency ^∗^ SOA interaction effect (i.e., the main effect of interest showing the results of reward on RTs) was not statistically significant: *F*(2,172.9) = 0.302, *p* = 0.74 (Figure 2). This result did not show any significant difference in the baseline vs. the test session between the different types of reward assigned to the participants.

##### ANOVA 4: with Bayesian approach

The parameters and methods used in this analysis were identical to those used in ANOVA 2, except for the use of BF_01_ instead of BF_10_, i.e., the Bayes Factor showing the likelihood of the data given H_0_ compared to H_1_, instead of the other way around. To this end, it is important to acknowledge that BF_01_ = 1/BF_10_.

This analysis showed that the effect of interest the time ^∗^ reward ^∗^ congruency ^∗^ SOA presented a BF_01_ = 14.493. This result indicated that data were fourteen times more likely to occur given the null than the alternative hypothesis. Therefore, we can state that this result shows a strong evidence for the H_0_, when considering different effects of the reward factor on RTs.

Given that our predictions were not confirmed by the analyses performed so far, we tested two other alternative outcomes.

First, we tested whether during the learning phase monetary reward changed the spatial priority map creating an attentional bias toward the rewarded side in the test phase compared to the baseline phase, independent of gaze direction. If this hypothesis was correct, we might expect that RTs for the reinforced spatial side should be faster independent of gaze-direction.

Second, we tested whether spatial side and gaze direction might interact, by reinforcing or decreasing their individual effects. In this case, an interaction between the rewarding gaze direction and the spatial location of the target stimulus should emerge.

### Data Analysis on Alternative Outcomes

To test the first alternative outcome we ran two more ANOVAs (the fifth and the sixth), using once again both classical (fifth analysis) and Bayesian (sixth analysis) hypothesis testing. Both analyses took into account two within-subject independent factors: time (2 levels: baseline session vs. test session), and side (2 levels: target appearing on the left vs. right side); and one between-subject factor: conditions (3 levels: condition A, condition B, and control condition), in a full factorial model. The dependent variable was the RTs averaged across all correct trials in each condition. The two SOAs were analyzed separately, since in the previous ANOVAs a statistically significant main effect of SOA was found, but no significant interaction effects involving this factor (see “Results” section). For this reason, RTs obtained for 250-ms SOA condition and for 750-ms SOA were split and analyzed separately.

The seventh and eighth ANOVAs aimed to investigate whether the rewarded spatial side and the rewarding gaze-direction interacted, using both classical analysis (seventh) and Bayesian analysis (eighth). Both analyses took into account three within-subject independent factors: time (2 levels: baseline session vs. test session), side (2 levels: target appearing on the left vs. right side), and congruency (2 levels: congruent vs. incongruent); and one between-subject factor: conditions (3 levels: condition A, condition B, and control condition), in a full factorial model. The dependent variable was the RTs averaged across all correct trials in each condition. These two further analyses were done both for 250-ms SOA and for 750-ms SOA separately for the reason discussed above.

#### Results

##### ANOVA 5: effects of reward on target side with frequentist approach

**FIGURE 3:**
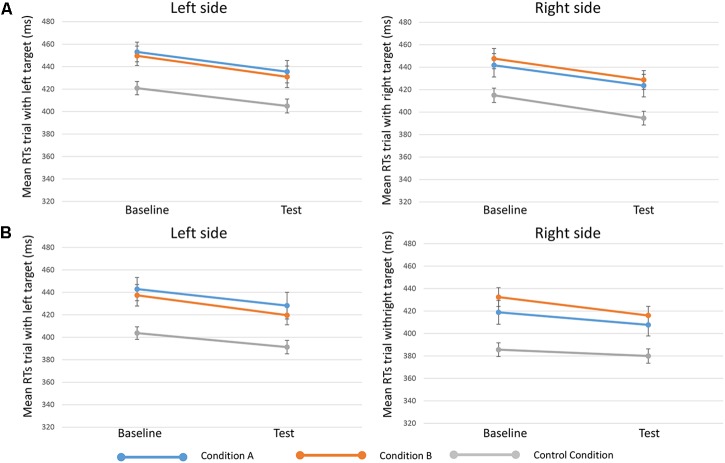
Mean RTs of trials when targets appeared on the left (in the left side of the figure, or “Left side”) and targets on the right (in the right side of the figure, or “Right side”) in the three conditions (condition A, condition B, and control condition)”. Upper in the figure **(A)** results for 250-ms SOA are represented, lower **(B)** results 750-ms SOA are showed. Bars represent the standard error.

Results from the fifth analysis demonstrated that the time ^∗^ side ^∗^ condition interaction effect (indicating the effect of reward on specific target sides) was not statistically significant either for 250-ms SOA [*F*(2,81) = 0.203, *p* = 0.817, $\eta_{p}^{2}$ = 0.005] or for 750-ms SOA [*F*(2,80) = 0.178, *p* = 0.838, $\eta_{p}^{2}$ = 0.004] (Figure 3).

##### ANOVA 6: effects of reward on target side with Bayesian approach

The sixth analysis showed that the time ^∗^ side ^∗^ condition interaction effect presented a BF_01_ = 14.93 (250-ms SOA) and a BF_01_ = 15.87 (750-ms SOA). This result indicates that data were about 15 times more likely to occur given the null rather than the alternative hypothesis, thus providing strong evidence (with both SOAs) for H_0_ (Lee and Wagenmakers, 2013), when considering different effects of reward on different target sides.

##### ANOVA 7: interaction between spatial side and gaze direction with frequentist approach

**FIGURE 4:**
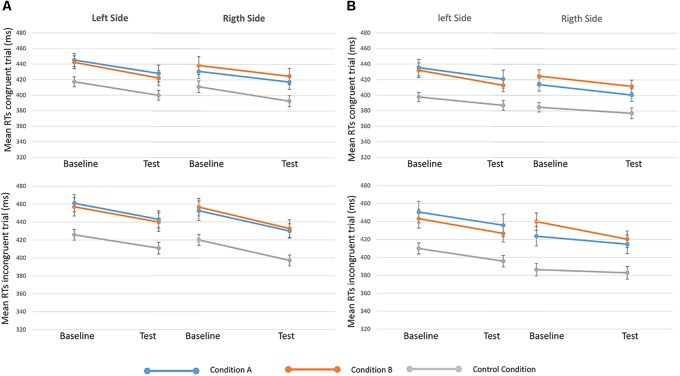
Mean RTs of congruent (upper in the figure) and incongruent (lower) trials in baseline and test sessions for the three conditions (condition A, condition B, and control) are showed, separated when targets appeared on the left and right sides. On the left of the figure **(A)** 250-ms SOA are represented, on the Right **(B)** 750-ms SOA are showed. Bars represent the standard error.

Results from the seventh analysis demonstrated that the time ^∗^ side ^∗^ condition ^∗^ congruency interaction effect (indicating the effect of reward on GCE) was not statistically significant either for 250-ms SOA [*F*(2,81) = 0.135, *p* = 0.874, $\eta_{p}^{2}$ = 0.003] or for 750-ms SOA s [*F*(2,80) = 0.647, *p* = 0.526, $\eta_{p}^{2}$ = 0.016] (Figure 4). These results show that H0 (i.e., no effect of reward on the GCE) cannot be rejected by using a frequentist approach. For this reason, we investigated the data also using a Bayesian approach.

All the main effects were statistically significant: SOA = 250 ms: time: *F*(1,81) = 49.638, *p* < 0.001, $\eta_{p}^{2}$ = 0.380; side: *F*(1,81) = 11.439, *p* = 0.001, $\eta_{p}^{2}$ = 0.124; congruency: *F*(1,81) = 65.018, *p* < 0.001, $\eta_{p}^{2}$ = 0.445; conditions: *F*(2,81) = 5.308, *p* = 0.007, $\eta_{p}^{2}$ = 0.116. SOA = 750 ms: time: *F*(1,80) = 21.635, *p* < 0.001, $\eta_{p}^{2}$ = 0.213; side: *F*(1,80) = 50.369, *p* < 0.001, $\eta_{p}^{2}$ = 0.386; congruency: *F*(1,80) = 27.755, *p* < 0.001, $\eta_{p}^{2}$ = 0.258; conditions: *F*(2,80) = 6.818, *p* = 0.002, $\eta_{p}^{2}$ = 0.146.

##### ANOVA 8: interaction between spatial side and gaze direction with Bayesian approach

BF_01_ was used to test the evidence for the null hypothesis relative to the critical interaction effect of the previous analysis. This analysis showed that the time ^∗^ side ^∗^ condition ^∗^ congruency interaction effect presented a BF_01_ = 13.33 (SOA = 250 ms) and a BF_01_ = 3.16 (SOA = 750 ms). This result indicates that data were 13 times (or 3 times) more likely to occur given the null rather than the alternative hypothesis, thus providing strong (with SOA = 250 ms) or moderate (with SOA = 750 ms) evidence for H_0_ (Lee and Wagenmakers, 2013), when considering different effects of reward on the GCE.

## Discussion

The present study aimed at understanding a possible interplay between GCE and reward processing, since, to the best of our knowledge, this has not been previously investigated. Previous studies, in fact, focused only on the motivational, thus implicit, role of reward associated with non-social stimuli in guiding selective visual attention (Della Libera and Chelazzi, 2009).

We investigated whether or not the capability of reward to implicitly orient visual attention, as observed with non-social stimuli, could interact with gaze cueing. Specifically, whether or not reward presentation was able to modulate (i.e., compete or enhance) GCE. To this end, we built a baseline-test experiment in which participants carried out a gaze cueing task before and after a session designed to deliver (implicit learning task) or not (perceptual discrimination task) a monetary reward in one of the two spatial locations in which the gaze cue was looking at. Overall, the aim underlying this baseline-test study was to investigate whether or not the repeated earning of money in relation to a given gaze direction (cueing left or right) could modify the magnitude of the GCE in the test session compared to the baseline session.

We applied both a frequentist and a Bayesian approach in order to analyze our data in the case of null results.

On the one hand, the results of the baseline session showed the presence of GCE – i.e., faster responses in congruent trials when the target appeared in the gazed-at location – and that GCE was not significantly different among the three conditions (condition A, condition B, control condition). Furthermore, we observed faster responses for 750-ms SOA than for 250-ms SOA, whereas no RT differences between congruency and incongruent conditions as a function of SOAs was found. Therefore, our data confirmed the wider literature that considered gaze cue and, more generally, social signals as a source of crucial information for directing the observer’s attention and effectively interacting with other humans to adapt to our social environment (Baron-Cohen and Cross, 1992; Baron-Cohen et al., 1997; Driver et al., 1999; Frishen et al., 2007). Accordingly, the automatic orienting of attention mediated by social cues such as gaze direction have *per se* a strong rewarding (motivational) valence, given its importance in providing the observer with relevant information about an event or a situation (e.g., about others’ mental states or emotions, possible dangers etc.). For this reason, the term “social motivation” has been coined to define a set of psychological dispositions, which also include gaze perception and gaze following. The perception of gaze direction are thought to be biologically determined and bias human beings to automatically and preferentially orient to the social world as well as to seek and maintain social bonds with other people (Chevallier et al., 2012).

According to our predictions, we expected that monetary reward influenced GCE as measured by incorrect responses or modulation of RTs.

In contrast, but no less interesting, in the present study we did not find evidence of a modulation in GCE following rewarding gaze direction as compared to non-rewarding gaze direction. Moreover, no effect was found for rewarded space location and no significant interaction between gaze direction and space location emerged either.

These findings highlight that, contrary to what was observed in previous attentional studies with non-social stimuli (Pessoa and Engelmann, 2010; Chelazzi et al., 2013; Sali et al., 2014), monetary reward associated with gaze direction (rewarding vs. no-rewarding) does not modulate (by enhancing or interfering) the orienting of attention toward a cued (looked-at) location as measured by GCE.

To date monetary reward and GCE have been separately studied although both are thought to have a significant motivational valence. Rewarding stimuli were reported to play an important role in prioritizing processing by deploying attention (Sali et al., 2014). Particularly, previously rewarded stimuli tend to automatically capture attention. This effect has been interpreted to be due to a learning process through which the presentation of a reward increases the motivational valence of a stimulus that *per se* had little or no motivational saliency (Chelazzi et al., 2013; Anderson et al., 2014). This learning process, indeed, would act by automatically allocating attentional resources only on target features (i.e., color) carrying information about the possibility to gain a subsequent reward by virtue of experiences (Sali et al., 2014).

Our findings clearly show that the effect of gaze direction in automatically orienting attention is not affected by monetary reward. In other words, it is plausible that the monetary valence of the reward is not relevant enough to either mitigate or enhance the attentional orienting effect of such a powerful social signal. Although researchers in economy and psychology supported that a commodity such as money without a biological valence in itself can nevertheless become a strong motivator (Lea and Webley, 2006), the motivation to obtain money does not have direct adaptive meaning and it does not result from an evolutionary process begun at human birth (Lea and Webley, 2006). It develops throughout the lifespan and through the interaction with the external and modern environment, providing the individual with information about the value of money. For this reason, monetary reward could be less powerful than gaze direction in automatically capturing and orienting attention in humans. On the contrary, orienting attention in the same direction of an averted gaze has the benefit of providing not only crucial information about the world around but also of informing us about the mental states of others (Baron-Cohen and Cross, 1992; Baron-Cohen et al., 1997; Driver et al., 1999). From an evolutionary point of view, social motivation would be at the basis of an adaptive tendency of the individuals to cooperate and create supportive environments (Rilling and Sanfey, 2011). The relevance of social motivation is also highlighted if we consider that the lack of it represents a core feature of some neuropsychiatric conditions, which are characterized by an impairment and strong difficulties in social communication and interaction such as Autism Spectrum Disorders (Chevallier et al., 2012). Nevertheless, evidence coming from studies, which examined gaze processing in Autism Spectrum Disorder (ASD), seems to be controversial as some studies showed that sensitivity to eye gaze is not atypical in this population (Leekam et al., 1998; Nation and Penny, 2008). However, other evidence comes from psychiatric research done on schizophrenic patients who present severe social-cognitive deficits (Mier and Kirsch, 2015). These patients were reported to be impaired in processing information conveyed by eye gaze (Tso et al., 2012; Dalmaso et al., 2013), but showed a normotypical performance in pointing tasks and in orienting of attention in response to arrow cues (Akiyma et al., 2008; Dalmaso et al., 2013).

The absence of a modulatory effect of the monetary reward could be also explained by the different tasks used in studying attentional orienting. For instance, in previous studies, a monetary reward was given as feedback after a correct response (Chelazzi et al., 2013). In our task, instead, the monetary reward was repeatedly associate not to the participant’s performance but to the contingence between a visual stimulus (the eye gaze cue) and the delivery of a reward. Therefore, it is also possible that a much higher number of associations between monetary reward and the gaze cue than that used in the present study is needed to better consolidate the memory trace at the basis of “the law of effect,” thus allowing the modulatory effect of monetary reward on GCE to emerge. This issue could be investigated in future research, for example, by increasing the number of times in which the reward is presented in the learning phase (Della Libera and Chelazzi, 2009).

Another possible interpretation of the null effect found in the present study might be that the automatic nature of GCE and/or the biological valence of gaze direction has determined its imperviousness to higher order motivational effects (i.e., monetary reward). However, as reported in the introduction, the full automaticity of the GCE has been questioned by a consistent number of studies. These studies showed that the orienting of attention induced by gaze direction can be modulated by top-down processes (Bayliss et al., 2010; Teufel et al., 2010; Wiese et al., 2012, 2013, 2014; Ehrlich et al., 2014; Wykowska et al., 2014; Cole et al., 2015; Perez-Osorio et al., 2017; Hayward and Ristic, 2018).

The present study has some limitations. One concerns the subjective valence that participants attributed to monetary reward. We did not systematically check if personality traits might have determined the null effect. Individual differences seem to play a key role in modulating the sensitivity and the relevance that participants attributed to monetary reward, as stated by the Reinforcement Sensitivity Theory of Personality (Corr, 2008). However, most of the aforementioned studies investigating the effect of monetary reward on attentional orienting, with non-social stimuli, did not take this inter-individual variability into account (Chelazzi et al., 2013 for a review).

Another limitation concerns the lack of a manipulation check assessing whether participants were able to learn the association between reward and gaze direction.

Indeed, the lack of interaction among time, side and condition does not allow us to conclude whether the association between gaze and reward was learned or not by participants, but leads to two possible explanation: on the one hand, it is possible that participants did not learn the association (gaze/reward). On the other one, it could be that learning took place, but gaze cues were so powerful in orienting attention that they win the motivational allocation induced during the learning phase through the reward.

Previous studies using the classical conditioning procedure (Pavlov, 1927), suggested that the passive presentation of stimulus-reward pairing is sufficient to induce implicit learning of the contingence between stimulus and reinforcement (Büchel et al., 1998; Pessiglione et al., 2008; Seitz et al., 2009). Together with evidence showing that the reward is a powerful way to allocating attention (e.g., Chelazzi et al., 2013, 2014), we are more likely to lean toward the second explanation. Taken together, the current findings show that the modulatory effect of monetary reward on attentional orienting, typically observed in studies with non-social stimuli, does not emerge when attentional orienting is mediated by gaze direction, a cue with a strong biological and social valence. The present results further highlight the relevance and strength of gaze cueing for humans. However, more studies are needed to corroborate our findings and to drive more exhaustive conclusion on whether and how monetary reward can modulate GCE.

## Conclusion

The new and interesting aspects suggested by the present study is that, despite the capital accumulation has become probably the most prominent motivational drive of the Western society, the signals that we receive from others still remain a primary source of information, which cannot be hindered by a monetary incentive.

## Author Contributions

PR, LL, and EG conceived and designed the study. EG, AV, and JDA ran the experiments. AV and FB conducted the statistical analysis. JDA, EG, AV, FB, and PR prepared the draft. EG, JDA, LL, and PR jointly produced the final draft.

## Conflict of Interest Statement

The authors declare that the research was conducted in the absence of any commercial or financial relationships that could be construed as a potential conflict of interest.

## Footnotes

1 http://www.pstnet.com
